# Value‐Based Experiences Related to Digital Follow‐Up Services Among Critical Care Survivors: An International Qualitative Study

**DOI:** 10.1111/nhs.70135

**Published:** 2025-05-20

**Authors:** Anna Zacharelou, Radenka Munjas Samarin, Karolina Mikulcic, Ana Brcina, Adriano Friganovic, Christina Jones, Peter Nydahl, Margo M. C. Van Mol

**Affiliations:** ^1^ Department of Intensive Care Adults, Erasmus MC University Medical Centre Rotterdam Rotterdam the Netherlands; ^2^ SmartUp d.o.o Zagreb Croatia; ^3^ Department of Anesthesiology, Postoperative Care, and Intensive Medicine in Gynecology and Obstetrics University Hospital Center Zagreb Zagreb Croatia; ^4^ Department of Quality Assurance and Improvement University Hospital Centre Zagreb Zagreb Croatia; ^5^ Department of Nursing University of Applied Health Sciences Zagreb Zagreb Croatia; ^6^ Department of Nursing Faculty of Health Studies Zagreb Croatia; ^7^ ICUsteps Charity London UK; ^8^ Nursing Research, University Hospital of Schleswig‐Holstein Kiel Germany; ^9^ Institute of Nursing Science and Development Paracelsus Medical University Salzburg Austria

**Keywords:** digital support, E‐health, health‐related quality of life, ICU follow‐up service, intensive care unit, postintensive care syndrome

## Abstract

Intensive care units (ICUs) are increasingly striving to provide cost‐effective and value‐based support. To meet this trend, digital solutions might offer appropriate opportunities for delivering remote, personalized follow‐up services. However, it remains unclear whether digital solutions align with survivors' preferences to improve post‐ICU quality of life. The aim was to explore the value‐based experiences related to digital follow‐up services among critical care survivors. A qualitative design, with focus group interviews from May through October 2023, was conducted with critical care survivors recruited from four European countries. A thematic approach was used to analyze the data. Twenty‐two participants were included, of whom half were women (*n* = 11). Three main themes were categorized: (1) powerless and uncontrolled, a search for regaining life; (2) adequate digital information; and (3) the role of technology and appropriate functionalities according to users' wishes. Overall findings across the four European countries highlighted value‐based preferences such as personalized online information delivery, the possibility of e‐consults with healthcare professionals, and digital access to peer support.

Summary

- This study explores critical care survivors' value‐based experiences across four European countries for the development and use of a digital recovery pathway.
- Using patient‐reported experience measures could target valuable innovations in supportive ICU follow‐up care.
- According to users' preferences, a digital tool should include a simple interface, multi‐modal accessibility to accommodate their needs, informative materials, and a virtual space for communication with peers and healthcare professionals.

## 1 Introduction

Health service delivery for critically ill adult patients in the intensive care unit (ICU) has been extended in recent years beyond treatment during admission to include additional follow‐up and rehabilitation care (Rosa et al. 2019; Schofield‐Robinson et al. 2018). However, an optimal model and the evidence‐based benefits of these aftercare practices remain inconclusive (Dimopoulos et al. 2024). ICUs are increasingly striving to provide cost‐effective and value‐based support for survivors and their family members (Ostermann and Vincent 2023). The model of value‐based health care (VBHC) initiates a shift from provider‐centered norms toward a holistic and user‐centered approach, seeking to enhance quality of care while ensuring the equitable and sustainable use of health resources (Porter 2010; Hurst et al. 2019). A standardized description of VBHC is lacking (Schapira et al. 2020); however, a general attribute is its emphasis on healthcare users including their experiences and values in life. For the purpose of this study, “value” has been defined as the perspective of the individual stakeholder regarding the underlying elements of quality, service, outcomes, and experiences (Harrill and Melon 2021; van Staalduinen et al. 2022).

VBHC is an important part of high‐quality care and practice innovation in every ICU, involving both patients and family members as partners in shared decision making and the monitoring of health‐related outcomes that they consider important. Patient/person‐reported outcome measures (PROMs) and patient/person‐reported experience measures (PREMs) seek to prioritize and standardize meaningful outcomes for the target group (Kingsley and Patel 2017; Kynoch et al. 2021). Thus, using PROMs and PREMs could drive valuable innovation in supportive ICU follow‐up care.

## 2 Background

Hospitalization in an ICU can have effects on critically ill patients after discharge, as they may experience short‐term or long‐term declines in quality of life (Alasad et al. 2015; Kim et al. 2023). Within a year after discharge, ICU survivors may develop new or worsening physical, cognitive, and psychological impairments, collectively known as postintensive care syndrome (PICS), which can persist beyond acute care hospitalization (Needham et al. 2012, 2013; Geense et al. 2021; Herridge and Azoulay 2023; Harvey and Davidson 2016). Physical impairment in PICS often includes ICU‐acquired neuromuscular weakness (Hermans et al. 2014; Fan et al. 2014; Bakhru et al. 2018), while cognitive impairments in attention, concentration, and memory, as well as psychological symptoms of posttraumatic stress disorder (PTSD), anxiety, and depression (Wade et al. 2013; Righy et al. 2019) have been reported frequently. The experiences and perceived deterioration in quality of life among ICU survivors and their relatives, while returning home and finding a way to recovery, have been extensively reported elsewhere (Seppänen et al. 2019; Cha and Ahn 2019; Goddard et al. 2024).

Easily accessible, comprehensive, and practical information, alongside personalized support for survivors and their family members throughout the recovery trajectory, has been proven essential in preventing adverse outcomes (Scheunemann et al. 2011). A structured follow‐up service is strongly recommended for critical care survivors (Renner et al. 2023), although its implementation in practice can be unavailable, costly, and most effective when provided by trained ICU professionals (Colbenson et al. 2019). ICU follow‐up services could therefore advance their care by offering easily accessible digital health programs (Dimopoulos et al. 2024; Berger et al. 2025).

The rapid transition to digital healthcare has increased acceptance of these services (McBride et al. 2021), enabling individuals to self‐manage their care at the time and place of their choosing (Ledel Solem et al. 2019; Moock 2014). Digital technologies may offer many advantages and opportunities for innovation in delivering healthcare. New digital technologies support health‐related activities (World Health Organization (WHO) 2019) and offer multiple delivery modalities such as remote consultations via mobile health apps and wearables, opportunities for providing information and supportive care, personal health monitoring, shorter waiting times, reduced travel costs for patients, and fewer face‐to‐face appointments for routine check‐ups (Fox et al. 2007; Hommel et al. 2013; Potluka et al. 2023; Kruse et al. 2017). Several clinical specialties, such as the mental health care sector (McBride et al. 2021) and cardiology (Wagenaar et al. 2019), have provided evidence of the efficacy of digital services. Additionally, cancer survivors, post‐discharge stroke patients, and patients experiencing chronic pain have expressed their desire for digital health support (Jansen et al. 2015; Davoody et al. 2016). The potential effectiveness of digital care and patients' reported preference for contact with healthcare professionals could warrant additional digital support in the ICU (Rose and Cox 2023). New initiatives in this domain have been launched, such as a tele‐rehabilitation with videoconferencing, virtual reality cognitive stimulation, and a self‐directed web‐based educational program (Cox et al. 2023; Capin et al. 2022; Gerber et al. 2019). However, it remains unclear whether digital solutions align with patient and family preferences to improve post‐ICU quality of life (Berger et al. 2025). Identifying which innovations are most valuable from the perspective of this target group regarding the underlying elements of quality, service, outcomes, and experiences is crucial for ensuring value‐based solutions for a digital transition in healthcare.

## 3 Objective

The objective of this study was to explore the value‐based experiences related to digital follow‐up services among critical care survivors in four European countries: Croatia, Germany, the Netherlands, and the United Kingdom.

## 4 Materials and Methods

### 4.1 Design

An exploratory design was used, and qualitative focus group interviews were conducted indicating value‐based experiences related to digital follow‐up services among critical care survivors. This study is part of a broader research program aiming at developing a digital follow‐up service for ICU survivors and family members (van Mol et al. 2023). Focus groups are an appropriate scientific approach for the in‐depth exploration of the context of specific topics described as PREMs, including digital ICU follow‐up services and related expectations among participants. The Consolidated Criteria for Reporting Qualitative Research (COREQ) checklist was used for reporting the study (Tong et al. 2007).

### 4.2 Setting and Participants

Adult ICU survivors were recruited from the ICUs of two hospitals; one in Croatia (HR) and one in the Netherlands (NL), and two patient self‐help groups running peer support with volunteers: Sepsis‐Help in Germany (DE), and ICUsteps in the United Kingdom (UK). Convenience sampling was used in all settings to recruit participants. Two approaches were utilized: (1) expert sampling, in which participants with expertise based on their own ICU experience were recruited by the local study coordinators and (2) self‐selection sampling, where individuals voluntarily participated based on predefined inclusion and exclusion criteria (Etikan and Bala 2017; Berndt 2020).

The inclusion criteria were as follows: an ICU stay exceeding 48 h, age 18 years or older, proficiency in the respective local language for effective communication and comprehension of study information, and general physical, mental, and cognitive stability. The stability criterion considered participants' perspectives, ensuring that they felt ready to participate, recall, and interact with others about their ICU experience. Participants with prehospital psychological or cognitive impairments were excluded from participation in the study. To maximize diversity, no predefined selection was made based on sex, level of education, or cultural background.

Eligible former ICU patients were invited to participate between May and October 2023 by the local study coordinators (A.F., C.J., M.M.C.V.M., P.N.) through different methods:

- In the Netherlands (NL): during regular inpatient follow‐up appointments,
- In Croatia (HR): via telephone calls 3 months after discharge,
- In Germany (DE) and the United Kingdom (UK): via mass email distribution.

Interested individuals received a written patient information brochure outlining the study aims and procedures. In HR and NL, participants signed a printed informed consent form at the beginning of the focus group interview, while in DE and UK participants received and returned a digital consent form prior to the scheduled focus group meeting. Participation was voluntary and confidential, with no incentives provided. Refusals were not archived. Participants were assured of their anonymity, the voluntary study approach, and their right to withdraw at any time, especially if they felt emotionally overwhelmed due to unpleasant memories or flashbacks of the ICU. No one dropped out.

### 4.3 Materials

An a priori script for the focus groups was developed with open‐ended questions, including an introduction on PICS and ICU follow‐up, continued with needs and preferences on digital applications in ICU recovery (Supplemental File 1). This semistructured interview guide offered the opportunity to elicit open responses while ensuring that relevant topics were assessed (Kvale and Brinkmann 2009). The guide was developed in Dutch and thereafter translated into Croatian, English, and German by professional language services. A pilot focus group was established in the Netherlands to determine the feasibility and the timeline of the interview structure.

### 4.4 Procedures

Qualitative data were collected in four focus group meetings, one in each participating country. These meetings were conducted in‐person (NL), online (UK, DE), or in a hybrid format (HR). All the focus groups had dual moderation, that is, they were facilitated by both the local study coordinators (A.F., C.J., M.M.C.V.M., P.N.) and an additional moderator, at least one of whom was experienced in conducting qualitative study methods. Moreover, the moderators (a mix of females and males) possessed backgrounds in ICU nursing, medicine, or psychology, held several degrees varying from bachelor to PhD, and were sensitive to the vulnerability of critical care survivors. The local language was used. All focus group interviews were audiotaped, transcribed verbatim, and then translated into English (where needed) for the purpose of analysis. Only the local study coordinator had access to the data via a password‐protected file. An independent company handled the translation, assigning professional translators fluent in both the respective local language and English. The study coordinators were accessible for cultural and linguistic clarifications.

### 4.5 Data Analysis

A thematic analysis of data was conducted according to Braun and Clarke in six steps (Braun and Clarke 2014): familiarization with the data; generating initial codes; searching for themes; reviewing themes; defining and naming themes; and producing the report. These data were organized in small and meaningful codes one line at a time, in an inductive manner without trying to fit them into a preexisting coding frame or without trying to make them align with the researcher's analytic preconceptions. Recurring patterns and meanings in the data were identified, thus enabling flexible and comprehensive interpretation of similarities and variances between countries. The entire process of analysis was performed by two independent researchers who were experts in qualitative methods and analysis (R.M.S., K.M.). Two additional researchers experienced in ICU care were involved in the initial coding list for the Dutch focus group (A.Z., M.C.M.V.M.). While employing the same six‐step thematic analysis process across all focus groups, a primary thematic framework was established by the initial focus group analysis and was used to guide the naming of the main themes and the subsequent analyses. During the analysis process, we followed dynamic back‐and‐forth reflections with the core research team. After each focus group, we developed and refined the coding until a framework, encompassing all elements, was established. This thematic approach ensured the maximization of common topics while acknowledging country‐specific differences in content. We validated this framework by incorporating expert opinions from the entire research team during regular online meetings and by reviewing existing literature. Ultimately, the research team agreed on the reliability of the codes and identified the themes as understandable and recognizable (Duran et al. 2006; Nowell et al. 2017). The themes and codes were final checked by the Dutch patient organization IC Connect, which ensured triangulation of evidence. Sample sizes were pragmatically determined to ensure adequate information power, which meant that we considered information richness relevant to answer the research question (Malterud et al. 2016).

## 5 Findings

### 5.1 Characteristics of Participants

A total of 22 participants participated in four focus group meetings of 1 h (DE, *n* = 5; HR, *n* = 5; NL, *n* = 6; UK, *n* = 6), half of whom were women (*n* = 11). The duration of ICU stay varied from 6 days to 9 weeks, and the time since discharge ranged from a few months to 10 years (Table 1).

**TABLE 1:** Demographic characteristics of study participants.

Country of residence	Sex	Age (range, years)
The Netherlands (*n* = 6)	4 women, 2 men	39–65
The United Kingdom (*n* = 6)	2 women, 4 men	41–70
Croatia (*n* = 5)	2 women, 3 men	Not reported
Germany (*n* = 5)	3 women, 2 men	35–75

Abbreviation: *N* = number.

### 5.2 Thematic Similarities and Variances

**FIGURE 1:**
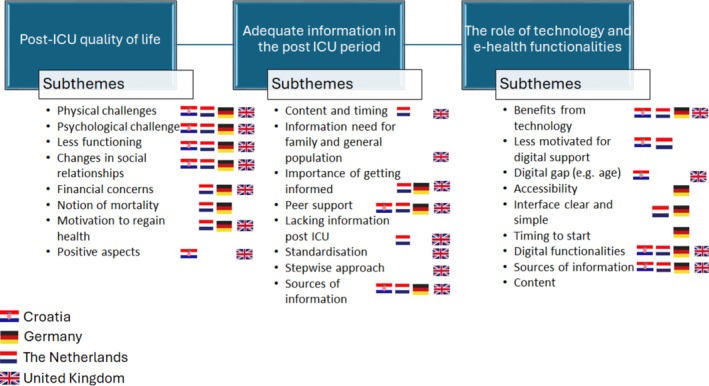
Main themes and subthemes identified in four European countries.

We identified three main themes: “Powerless and uncontrolled,” a search for regaining life; “Adequate digital information”; and “The role of technology and appropriate functionalities according the users' wishes.” Each main theme had several subthemes, 38 in total spread among the four countries (Figure 1). The patient reported experiences, as perspectives of the individual stakeholder regarding the underlying elements of quality and service in follow‐up, are described as exemplary quotes.

#### 5.2.1 Powerless and Uncontrolled, a Search for Regaining Life

The first theme, which was common to all countries, showed that quality of life in the post‐ICU period was significantly impaired for most of the participants. They reported having to face intertwined physical and psychological difficulties, primarily stemming from physical impairments that, at times, led to psychological consequences and emotional states hindering physical recovery. These experiences of being critically ill played an overwhelming and recurrent role in everyday life.

*Such a situation is traumatizing, and this has an impact also on the physical recovery*. (DE).

Participants experienced a quest for sufficient help, sometimes feeling powerless and without control over their own rehabilitation (NL). Additional reasons for frustration were a lack of understanding from family and society, as well as difficulties accessing medical care and professional help. This made participants feel lost and abandoned (DE, HR, NL). Digital consults to healthcare professionals and online peer support could make life easier. Increased financial needs for treatment and limited possibilities for work caused financial difficulties for some participants from HR, NL, and UK. Therefore, cheap and accessible recovery, such as with online help, would be most welcome.

*Not being able to return to work placed me in a financial straitjacket that I am yet to emerge from*. (UK)

ICU admission and changes in health conditions led to changes in family and social relationships in all countries. The participants reported difficulties in finding their place within their family and society again, as some survivors felt like a ballast to their families. A lack of understanding and inappropriate reactions to health conditions and changes in physical appearance led to a reduction in social contact. The experience of the seriousness of their health condition led to increased awareness and confrontation with one's own mortality for some patients in DE and NL. Participants reported lacking spiritual support in the post‐ICU period.

*You are so confronted with your own mortality, I didn't find it a very pleasant experience. I felt out of control*. (NL)

Despite obstacles and challenges in the post‐ICU period, participants showed motivation and made efforts to regain physical, emotional, and cognitive health (DE, HR, NL). They found inspiration mostly in their children and the desire to become as independent as possible again.

#### 5.2.2 Adequate Digital Information

Participants from NL and UK highlighted that their expectations of quality and service in ICU follow‐up varied across individuals and that the support should be tailor‐made. UK participants suggested that family members should also receive information so that they would know what to expect, how to adapt to the new situation, and how to best provide support to discharged patients.

*The education for other people is so lacking… I wish that people had understood it, because people still don't understand it now*. (UK)

Participants from NL, DE, and UK reported that being informed about what happened with them in the ICU was very important, especially if they were in a coma for the majority of their ICU stay. At this point, they mentioned the importance of having an ICU diary kept for them by their family members or by doctors/nurses in the hospital. Participants, for whom an ICU diary was not kept, felt frustrated and expressed resentment toward their family for not keeping one, while participants from DE with no ICU diary did not know where and how to access their personal patient data. A digital diary was reported as lacking.

*I missed time, but I was fortunate. I have an ICU diary that covers the first 550 days of my hospitalization and that has allowed me to anchor my experience in reality, I would be lost without it*. (UK)

Participants from NL and UK reported a lack of vital post‐ICU information and uncertainty about where to find reliable help. This information gap posed a significant obstacle for Dutch participants in accessing adequate professional support during their recovery process. Adequate digital alternatives could have been valuable.

*I don't know where to look for help if it's not offered. Without digital support it is such a vague and unstructured search*. (NL)

*I mean, if I wouldn't be here without ICUsteps [the digital support]. It's been absolutely a lifesaver … it was absolutely invaluable all the way through and I'm probably still looking at it now*. (UK)

Participants primarily sought useful information from the hospitals where they were treated. In NL and HR, some patients received excellent support and were given guidance on where to seek further help. The well‐informed participants also mentioned rehabilitation centers, post‐COVID outpatient clinics (NL), visiting nurses, emergency rooms (HR), and Facebook support groups (HR). However, not all participants found Facebook groups to be helpful (DE). For those without access to reliable sources, internet search engines were commonly used. Regarding existing materials, UK participants found it challenging to select relevant and trustworthy digital information due to the abundance available.

*There seemed to be an endless trail of googling and ringing people, mishmash of a mess of no information*. (UK)

Interaction with patients with similar experiences was important to participants from all countries; peer support could provide practical information and support that cannot be provided by those who have never experienced the same incidents, family members, or healthcare providers. Sharing the same experiences made participants feel that they were not alone.

*Fellow sufferers. I never knew about that. Before the interview started, we had already cried 2 times. I have never been able to talk to fellow sufferers. Here I can just cry. I think online stories would also help*. (NL)

*There's survivor stories on one of these sites I've been on … I found those really helpful, although they were nothing like my experience*. (UK)

#### 5.2.3 The Role of Technology and Appropriate Functionalities According to the Users' Wishes

**FIGURE 2:**
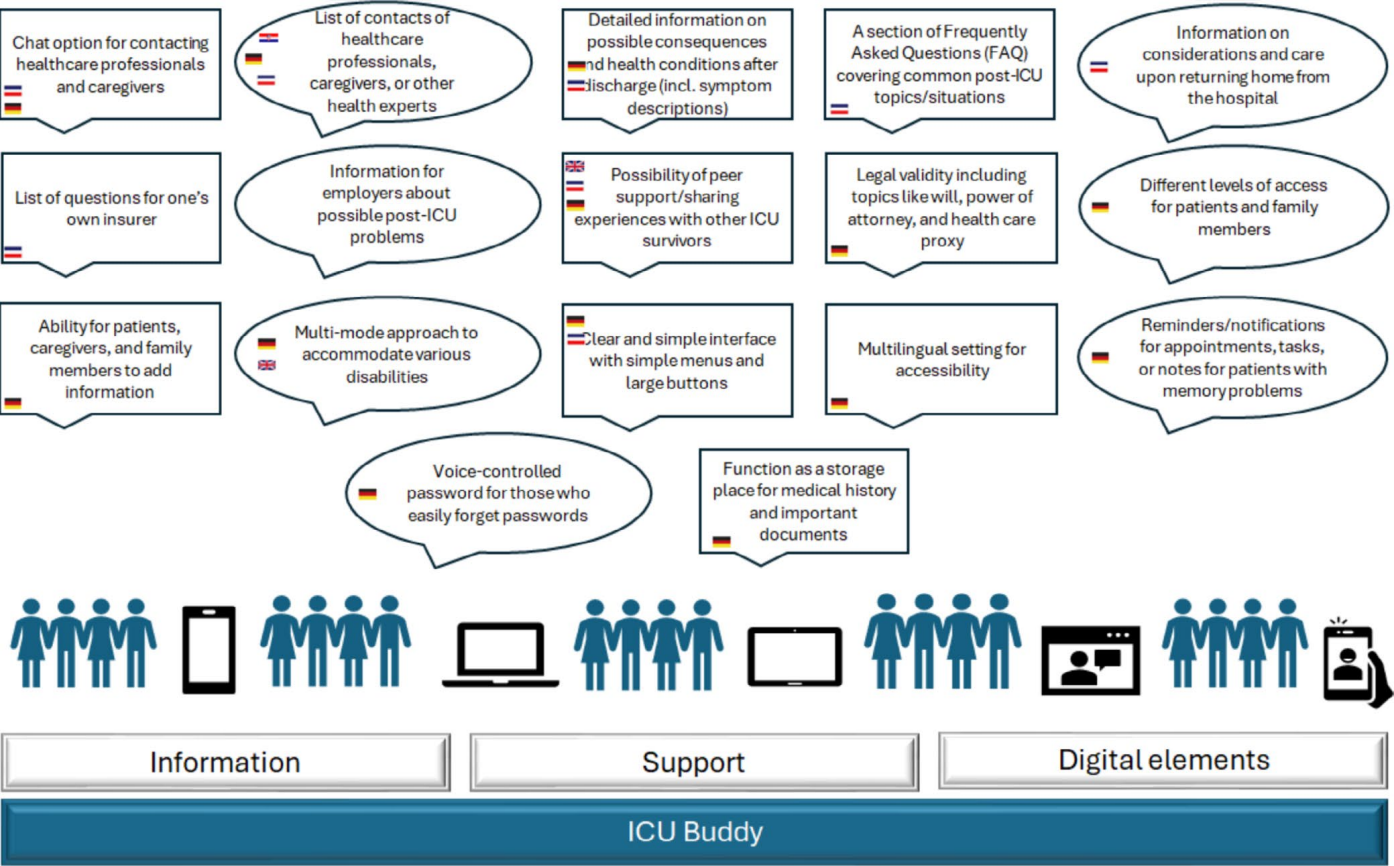
Expected functionalities of the e‐health tool in ICU follow‐up service: “The ICU buddy.”

Most participants acknowledged the potential benefits of technology in addressing information gaps and supporting digital solutions (Table 2). Some older participants in HR and NL expressed resistance to digital means and highlighted preferred digital support options such as contacting healthcare professionals via email, WhatsApp, or Zoom. Based on what they missed during their own recovery process, participants from DE and NL provided clear insights into the desired features and functionalities of a digital tool for post‐ICU support (Figure 2).

**TABLE 2:** Overview of exemplary quotes regarding digital ICU support.

The role of technology and suggested digital functionalities
1. Need for online contact with healthcare professionals Participants requested the possibility to contact healthcare professionals via online communication platforms.
*As long as you have a few contact people, maybe through the app, who you could contact perhaps, if necessary, if they agree to it, when you're not doing well*. (DE)
2. List of healthcare contacts Participants requested a list of contacts of healthcare professionals, whom they can contact.
*To even have a contact person who actually knows something about sepsis, who knows the symptoms and what it does to a person afterwards*. (DE)
3. Detailed information on post‐ICU health conditions Participants requested detailed information on possible consequences and health conditions that may appear after discharge.
*Should be able to find out in the first place what ailments, what special circumstances there could be, after a hospitalization in the ICU*. (DE)
4. Frequently asked questions (FAQ) section Participants requested a frequently‐asked‐questions (FAQ) section, which will cover common post‐ICU situations/topics.
*But I would have a frequently asked questions, a list of phone numbers with who do I call for this, who do I call for this*. (NL)
5. Guidance for transitioning home Participants requested information on what should be considered and taken care of, when returning home from hospital.
*Also information about coming home from hospital “what to think about*.*”* (NL)
6. List of questions for insurance providers Participants requested a list of questions for one's own insurer.
*And a list of questions for my insurer. Those are very practical basic things that as a nurse you don't necessarily have time for to give when someone leaves hospital. And those are things you run into that are inconvenient*. (NL)
7. Information for employers Participants requested information that can be given to the employers about one's own possible problems.
*What can I tell my employer about how to deal with me and all the problems that come with it*. (NL)
8. Access to peer support Participants requested the possibility of peer support.
*There's survivor stories on one of these sites I've been on … I found those really helpful, although they were nothing like my experience. People just talking like this now and saying just makes you think, well, I'm not the only one*. (UK)
9. Option to add personal notes Participants requested the possibility to add information.
*To be able to write down: how am I doing on that day, what happened during that day, the events so to say*. (DE)
10. Storage for medical records Participants requested that the tool should serve as a storage place for medical history and important medical documents.
*Maybe you could also scan a document and save it there, where it has a place for such things. Something where you could easily save documents perhaps, photos or PDFs*. (DE)
11. Reminders and notifications for important appointments and tasks Participants requested reminders or notifications for important appointments or tasks, as well as the ability to make notes (written or verbal), which would be very helpful for patients with memory problems.
*It should remind me. Of tasks, for example*. (DE)
12. Legal aspects: will, power of attorney, and healthcare proxy Participants requested that the tool have legal validity and include topics such as a will, power of attorney and health care proxy.
*The topic of a will, patient's directive*, etc. *Power of attorney, health care proxy, it's a huge subject. Yes, if you've survived such a situation, at the latest then you think about these things*. (DE)

Participants suggested: a list of contacts of healthcare professionals whom they can contact; a Frequently‐Asked‐Questions (FAQ) section; which will cover common post‐ICU situations/topics; detailed information on possible consequences and health conditions that may appear after discharge; information on what should be considered and taken care of when returning home from hospital; a list of questions for one's own insurer; and information that can be given to the employers about one's own possible problems.

*Should be able to find out in the first place what ailments, what special circumstances there could be, after a hospitalization in the ICU*. (DE)

*But I would have a frequently asked questions, a list of phone numbers with who do I call for this, who do I call for this*. (NL)

Other proposed functionalities were a chat option for contacting healthcare professionals and caregivers; a platform for sharing experiences with other ICU survivors; the ability for patients, caregivers, and family members to add information; a storage place for medical documents; and reminders or notifications for appointments, tasks, or note‐taking for those with memory problems.

*Maybe you could also scan a document and save it there, where it has a place for such things. Something where you could easily save documents perhaps, photos or PDFs*. (DE)

In particular, the German participants expressed concerns about the legal validity of digitally documenting a personal will, the power of attorney, and the healthcare proxy access.

The topic of a will, patient's directive, power of attorney, health care proxy, it's a huge subject. Yes, if you've survived such a situation, at the latest then you think about these things. I would have liked a digital checklist and a safe place to store. (DE)

A clear and simple interface for the digital tool, with simple menus and large buttons, following a click‐through model, a multimode approach to accommodate various disabilities, different levels of access, a voice‐controlled password functionality, and a multilingual setting is how participants imagined the digital tool. Participants from NL also came with a name suggestion.

I do think it's a nice name. An ICU buddy. What a good one. You could also call the app that. (NL)

## 6 Discussion

Value‐based experiences, measured as patient reported experiences among critical care survivors, reflected the needs and preferences regarding digital follow‐up service. Three main themes were categorized: (1) powerless and uncontrolled, a search for regaining life; (2) adequate digital information; and (3) appropriate functionalities according to the users' wishes. Seeking to identify critical care survivors' expectations and preferences for the use of a digital health tool is a crucial aspect of value‐based ICU follow‐up services.

Study findings showed that quality of life significantly declines after an ICU stay, as many practical challenges at home were amplified by physical and psychological difficulties. Previous studies have extensively reported a similar decline in quality of life, loss of independence, and an increased need for assistance after ICU discharge (Herridge and Azoulay 2023; Cox et al. 2023; Griffiths et al. 2013; Kean et al. 2017). Surviving a critical illness needs a personalized ICU support service, as transitioning through recovery is not a scheduled care path (Kean et al. 2017). Value‐based digital interventions could meet ICU survivors' way and time to recovery. Emotional reactions during the focus group sessions were evident, confirming that the ICU environment provokes a range of adverse psychological reactions in patients (Svenningsen et al. 2017). Participants felt abandoned and powerless when accessing personalized medical services, struggled to find their place and role in family and society, and had trouble returning to their previous level of functioning in life. Digital support in ICU follow‐up cannot address all the challenges survivors face. However, easily accessible information and digital support to stimulate self‐management could align with some of their needs. These informational and support needs change over time (King et al. 2019; Czerwonka et al. 2015). Therefore, a value‐based ICU follow‐up service should be designed based on critical care survivors' reported priorities in several time frames, focusing on empowering them to take control and strengthening personalized goal‐setting approaches.

Digital technologies are still encountered with some skepticism, especially among the older generation (Leung and Chen 2019). Although some individuals independent of age expressed resistance, most participants in our study were generally in favor of using a digital health self‐management intervention for ICU follow‐up support. A scoping review on digital health use among elderly people showed that despite some barriers, such as a lack of self‐efficacy and knowledge, active engagement of the target end users in the design and delivery of digital health programs is a key facilitator (Wilson et al. 2021). Some promising initiatives for a digital ICU recovery path have recently been launched (Rose et al. 2022; Gawlytta et al. 2022; Kanschik et al. 2023; Shin et al. 2023). The findings from our study clearly add the perspectives of the target group to these initiatives.

Insights mainly from Germany and the Netherlands emphasized the preferred features of digital tools, including contacting healthcare professionals and retrieving comprehensive online post discharge information. Similarly, improved communication between patients and hospital teams, guidance on follow‐up care, and the importance for consultants to share their contact details have been recommended previously (Castro‐Avila et al. 2021). However, participants in our study emphasized their need for tailored information delivery beyond a general written brochure, better‐informed family members, and increased public awareness of the post‐ICU recovery process. These needs and preferences correspond to the findings of both Rose et al. (2022) and Cox et al. (Cox et al. 2023), thus emphasizing critical care survivors' desire for personalized content in digital means in follow‐up services. These digital innovations are still in its infancy, but could open new pathways to addressing perceived barriers among the vulnerable target group, such as physical and financial constraints using in‐hospital recovery programs (Leggett et al. 2024). Digital health applications can stimulate individuals to regain control, offering more than just static website reading by empowering them in decision‐making and self‐management of their health (Painter et al. 2023). Self‐management interventions are crucial for improving patient health outcomes as these could be helpful for patients with chronic diseases adapting to life changes and preventing worsening conditions (Huang et al. 2024). Therefore, among ICU survivors who experience long‐term symptoms of psychological distress, enhancing self‐management could support coping with life‐changing events related to their critical illness. With digital applications becoming an integral part of modern daily life—such as smart watches or other devices measuring health outcomes—interaction with digital health services in ICU follow‐up is expected to improve goal‐setting and self‐management (Eaton et al. 2024).

Being supported by peers and having the chance to talk about one's own health situation might prevent the deterioration of health related impairments (Ledel Solem et al. 2019). Sharing similar experiences via a chat option could become a major source of online community support. All these digital functionalities according to the users' wishes, are fundamental for developing new interventions in high‐quality care and practice innovation in the ICU.

Due to the different healthcare and rehabilitation systems, some cultural differences were encountered. Participants from the Netherlands experienced limitations in accessing professional support during their recovery process, while participants from the United Kingdom expressed challenges in selecting relevant and trustworthy information. Participants from both countries emphasized the importance of tailored information delivery, suggesting that it should be tailor‐made to individual needs and circumstances. Additionally, the sources of information and support, such as hospital guidance, rehabilitation centers, online support groups, and specific healthcare applications, varied among countries. With respect to legal validity, a digital ICU recovery pathway should comply with the General Data Protection Regulation (GDPR) and the local (privacy) laws of the country in which it is used (Verhoeven et al. 2022; Vokinger et al. 2020). Participants mentioned the need to safeguard their own privacy, since digital tools can collect sensitive personal data. Any new digital application should therefore strive for data transparency, where personal data are used with integrity, lawfully, fairly, and traceable for valid purposes.

A core component of VBHC is the focus on the most important outcomes and experiences that matter and make a difference to patients, the public, and society, using the best available evidence (Hurst et al. 2019). Therefore, measuring value varies depending on the context. Our study emphasizes the importance of considering ICU survivors' perspectives, thus, a core aspect of VBHC (Porter 2010; Zanotto et al. 2021). The findings of this study, as PREMs reported in a meaningful way to the target group, could offer insights for decision‐making processes to align future investment of resources allocated to develop digital applications in ICU recovery. This might fed back to financial costs in transparent considerations. A goal of value‐based care transformation is to enable the healthcare system to create greater value for patients (Teisberg et al. 2020). However, while healthcare organizations are becoming more patient‐centered, stating that patients' values and perspectives are taken into account, general quality benchmarks still measure overall hospital mortality, infection rates, and medication errors (Zanotto et al. 2021). Innovation processes may lack a thorough inquiry through measuring outcomes, such as PROMs, and experiences, such as PREMs, relevant to the target group (van Gemert‐Pijnen et al. 2011; Kip et al. 2025). With our findings, we contribute to knowledge on the innovations that are most valuable from the perspective of ICU survivors and meet value‐based solutions in the digital transformation of healthcare.

### 6.1 Strengths and Limitations

Our study has the potential to contribute to digital ICU follow‐up services, offering tailored support for patients and family members. The sample had large variability in diagnoses, age, years since ICU experience, and single or multiple ICU admissions, supporting rigor and validity. The insights gained from this study promote the adoption of digital care solutions, ensuring that support is provided at the right place and time, thereby alleviating the burden on the healthcare system. However, our study has some limitations. First, the recruitment of participants willing to discuss their ICU experience and needs in a digital focus of follow‐up might have biased the answers through an overly engaged study sample. This group may not reflect the wider critical care survivor population, many of whom may not be inclined or able to engage with digital solutions due to factors such as digital literacy, limited access to technology, or lack of interest. Consequently, the findings should be interpreted with caution. Second, recall bias among participants, due to a time gap of 10 years after ICU admission, might have influenced memories, worsening or improving their views on recovery. Third, the small sample sizes and settings in four European countries might limit data saturation. Choosing a suitable sample size in qualitative research is an area of conceptual debate and practical uncertainty *(*Vasileiou et al. 2018 *)*. Therefore, we used information power to indicate that the richness in our data warrants the lower number of participants. Another bias could be the difference in the modus of data gathering, online or in‐person, in particular in regard to the topic of interest. These factors may have impacted the rigor of the data collection; however, the participants' responses and interactions showed consistency in the content. With the aim of gathering meaningful answers, we carefully addressed all participants and received detailed and insightful opinions in all meetings (Rupert et al. 2017; Bozkurt 2028). Thus, the risk of bias was reduced and comparable to individual differences in group participation. Future research should address these limitations with larger, more heterogeneous samples to provide a more detailed understanding of critical care survivors' expectations regarding utilization of digital follow‐up services. Fourth, the design of our study was not appropriate for international comparisons of country‐specific characteristics such as local digital infrastructure, digital awareness and competences, differences in healthcare systems, and current ICU follow‐up. This may limit the applicability of the findings, as digital interventions may need to be tailored to the unique healthcare environments and digital capabilities of each unique system. Therefore, future research should investigate the differences among international healthcare systems and assess technological readiness and infrastructure in each country to support the implementation of a digital follow‐up health service.

## 7 Conclusion

Value based experiences among critical care survivors from Croatia, Germany, the Netherlands, and the United Kingdom were explored for their preferences and expectations regarding the development and use of digital ICU follow‐up service. General findings across the four European countries included personalized online information delivery, the possibility of e‐consults with healthcare professionals, and digital access to peer support. Although varying cultural and systemic differences among countries could not be addressed, the critical care survivors' experiences might be key in future value‐based initiatives for developing digital applications in ICU recovery pathways.

## 8 Relevance for Clinical Practice

VBHC covering health service delivery and quality improvement in the ICU environment and rehabilitation services needs to respect, value, and understand how survivors may perceive the different aspects of the aimed innovation. Outcomes are measurable with PROMs, such as symptoms of PICS‐F and quality of life, and PREMs, such as personal experiences and satisfaction scores. Using PROMs and PREMs could also target valuable interventions in supportive ICU follow‐up care, including the development of digital health interventions. The needs and preferences of ICU survivors serve as the foundation for gaining insight into what exactly should be developed, built, tested, and evaluated (Zacharelou et al. 2024). Critical care survivors prioritized a digital tool with a simple interface, multimodal accessibility to facilitate their needs, informative material, and a virtual space for communication with peers and healthcare professionals.

## Author Contributions

**Anna Zacharelou:** investigation, writing – original draft, methodology, formal analysis, data curation, validation, visualization. **Radenka Munjas Samarin:** methodology, writing – review and editing, formal analysis, data curation. **Karolina Mikulcic:** writing – review and editing, formal analysis, validation. **Ana Brcina:** investigation, writing – review and editing, validation. **Adriano Friganovic:** investigation, validation, writing – review and editing, conceptualization. **Christina Jones:** investigation, validation, writing – review and editing, conceptualization. **Peter Nydahl:** investigation, validation, writing – review and editing, conceptualization. **Margo M. C. Van Mol:** conceptualization, funding acquisition, writing – original draft, visualization, methodology, formal analysis, supervision, writing – review and editing, project administration, resources.

## Ethics Statement

This study was conducted according to the principles of the Declaration of Helsinki and in accordance with the Dutch Medical Research Involving Human Subjects Act. The Medical Research Ethics Committee of Erasmus Medical Center [MEC‐2022‐0153] [anonymised] granted approval for the study. Thereafter, Institutional Review Boards in the three participating countries waived from the requirement to provide insurance for subjects participating in medical research.

## Consent

The authors affirm that individual participants provided informed consent for publication of their data. No material from other sources has been used.

## Conflicts of Interest

The authors declare no conflicts of interest.

## Supporting information


**Data S1.** Supporting Information.

## Data Availability

Anonymized data gathered and analyzed during the study will not be available publicly due to legal and ethical restrictions. These will be freely available at a reasonable request to any scientist wishing to use them for non‐commercial purposes as well as text and photo material of the developed intervention. The results of the study will be disseminated to healthcare professionals, health services authorities, and the public via presentations at national and international meetings and published in peer‐reviewed journals.
