# Supplementary material for: Value‐Based Experiences Related to Digital Follow‐Up Services Among Critical Care Survivors: An International Qualitative Study
Source: Nurs Health Sci. 2025 May 20;27(2):e70135. doi: 10.1111/nhs.70135 (PMC12089899; doi:10.1111/nhs.70135)
Supplement: Supplementary file 1 — Data S1. Supporting Information. [file NHS-27-e70135-s001.pdf]

Topic list interview groups

The aim of the DIPA-study is to explore opinions and relevant aspects regarding an e-health intervention providing information and support in personalized aftercare among ICU survivors in four European countries.

The main question to be answered is:

What are the needs and priorities in ICU follow-up service among ICU survivors, regarding an e-health intervention providing digital information and support?

This question will be answered by three sub-questions:

- What aftercare was provided to ICU survivors and what are their experiences?
- Which aspects of the support for symptoms of the Post-Intensive Care Syndrome (PICS) are important in ICU aftercare? More specifically, what are the information and support needs in ICU follow-up services among ICU survivors?
- What do ICU survivors find most desirable when using an e-health intervention?

Introduction

- Welcome and many thanks for participation in this focus group meeting
- Introduce yourself shorty
- Explain the purpose of the interview in simple language
- Explain that a recording will be made and that results will be processed anonymously
- Explain that everyone has a valuable say, and that the moderator could have questions of shorten the discussion for sake of the study questions. It is not meant to be brute.
- If you get overwhelmed, feel anxious or want to get out of this room, please let us know. Someone will be there for you providing support that you need.

Questions

1. Opening question

- Please, start to tell us a bit more about yourself.
- How did you come into the intensive care unit, what happened?

2. Introductory question (not too difficult to answer)

- When you think back on the ICU period, what feelings and thoughts does it bring to your mind?

3. Transition question (Lead the conversation if necessary)

Note the time guard, this is to provide background and confidence in the beginning of the interview. It's not the main question. But sometimes there is a tendency of participants to tell a lot about this.

- Do you recognize physical symptoms in yourself? (In particularly after the ICU period) If so, what were they?
- Did you encounter mental (psychological) problems during or after the ICU admission, if yes, could you provide an example?
- What problems did you have socially (work, support from others, relationship)?

→ *Opening to aftercare*.

4. Key question 1 (Aftercare).

Introduction: Aftercare is offered by some agencies (for example, an aftercare clinic).

- Have you had experience with aftercare and how was this for you?
- What made it difficult to seek help for your own mental/physical complaints afterwards?
- Were there activities or processes that made it easy?
- Did you undertake online activities outside healthcare institutions (eg hospital) to support yourself, and if so, could you explain these?

5. Key question 2. (Needs and preferences)

Introduction. With your previously mentioned symptoms (of PICS) in mind *(summarizing what the participants said earlier)*, **what do you need in regard to information and dealing with these symptoms?**

- What information did you need after the ICU admission?
- Suppose we are going to offer information; at what point (during, afterwards, etc.) should we do that? And how can we do that best (provide some suggestions; brochures, by telephone, volunteer, professional, digitally, via the hospital, via GP, otherwise...)
- For which symptoms (physical/social/psychological) did you need support? (For example worrying)

This might give an opening to eHealth.

6. Key question 3. Opinions on E-health

(e-health to be developed, explaining on examples of e-health, brainstorming time)

- What is your opinion, or experience, with information or support online, e.g., a website, a personal health platform or an app?

- How could technology contribute to IC aftercare?
    - o Why/why not?
- How should the digital support be designed (website, app)?
- What would you like to see reflected in the e-health tool?
- When would be the best time to offer the e-health application?
- Other ideas: how could we offer support in a different way?

Closing

We are coming to an end of this meeting.

- Is there anything you would like to add?

Provide a general summary and repeat the study procedure if necessary

- Thank you for participating

If participants have any concerns, provide a telephone number or refer them to a general practitioner, ICU aftercare or other professional assistance.
